# Minimally Invasive Surgery in Patients With Intracerebral Hemorrhage: A Meta-Analysis of Randomized Controlled Trials

**DOI:** 10.3389/fneur.2021.789757

**Published:** 2022-01-13

**Authors:** Duanlu Hou, Ying Lu, Danhong Wu, Yuping Tang, Qiang Dong

**Affiliations:** ^1^Department of Neurology, Shanghai Fifth People's Hospital, Fudan University, Shanghai, China; ^2^Department of Neurology, Huashan Hospital, Fudan University, Shanghai, China

**Keywords:** minimally invasive surgery, intracerebral hemorrhage, meta-analysis, death, craniotomy

## Abstract

**Background:** Minimally invasive surgery for intracerebral hemorrhage (ICH) has been evaluated in clinical trials. Although meta-analyses on this topic have been performed in the past, recent trials have added important information to the results of the comparison. However, little work has been done to compare the effect of MIS and conventional treatment on patient prognosis, especially mortality.

**Methods:** PubMed, EMBASE, Web of Science, Ovid, China National Knowledge Infrastructure, and ClinicalTrials.gov were searched on May 1, 2021, for randomized controlled trials of MIS for spontaneous ICH. The primary outcome was defined as death at follow-up, while the secondary outcome was defined as death in different comparisons between MIS and craniotomy (CT) or medication (Me).

**Results:** The initial search yielded 12 high-quality randomized controlled trials involving 2,100 patients. We analyzed the odds ratios (ORs) for MIS compared with conventional treatment, including Me and conventional CT. The OR and confidence intervals (CIs) of the primary and secondary outcomes were 0.62 (0.45–0.85) for MIS vs. conventional treatment. We also conducted subgroup analyses and found that the ORs and CIs for MIS compared with that of conventional treatment in the short-term follow-up were 0.58 (0.42–0.80), and, in the long-term follow-up, was 0.67 (0.46–0.98); and found that ORs were 0.68 (0.48–0.98) for MIS vs. CT and 0.57 (0.41–0.79) for MIS vs. Me.

**Conclusions:** This meta-analysis demonstrates that certain patients with ICH benefit in short- and long-term follow-up from MIS over other treatments, including open surgery and conventional Me.

**Systematic Review Registration:** https://www.crd.york.ac.uk/PROSPERO/.

## Introduction

Spontaneous intracerebral hemorrhage is the second most common subtype of stroke and is a critical disease with high mortality 2-6-fold higher than ischemic stroke (1, 2). Clot volume is the best predictor of outcomes regardless of the hemorrhage location (2, 3). Surgical therapies, including open craniotomy and stereotactic therapy, with or without thrombolysis, are beneficial in hemorrhage evacuation (4). However, in many patients, the surgical approach causes trauma to the surrounding brain in negating the benefit of hematoma evacuation. Thus, minimally invasive surgery (MIS) is the most promising surgical strategy for patients with ICH. MIS refers to surgery accomplished with a smaller incision and minimal surgical stress, and was first introduced in 1987, which performed the first laparoscopic cholecystectomy in history (5). The MIS for ICH can be performed using an endoscope or needle through a smaller incision, and a bone hole to suck the clot from the brain. Recently, randomized controlled trials (RCTs) have been performed to evaluate MIS in comparison to either medical therapy or conventional craniotomy with different surgical techniques (6). However, the conclusions are controversial; some studies showed the net benefit of MIS, while, in others, no benefit of MIS was observed. This may be due to the different study designs and ICH definitions (7, 8). Ongoing RCTs include two industry-sponsored trials: (1) the Early Minimally Invasive Removal of ICH trial sponsored by NICO Corporation, and (2) the Minimally Invasive Endoscopic Surgical Treatment with Apollo/Artemis in Patients with Brain Hemorrhage trial sponsored by Penumbra (9). Previous meta-analyses focused on the benefits of MIS over other therapies (10, 11), and found that MIS independently seems to decrease the rate of moderate-to-severe functional impairment and death at long-term follow-up. However, the selected data in these studies were doubtful, and the comparisons of mortality between patients who underwent MIS and those who underwent craniotomy were insufficient (10). Our study aimed to investigate the impact of MIS on ICH prognosis.

## Materials and Methods

### Study Design

We performed a systematic review and study-level meta-analysis of RCTs evaluating the ICH treatment according to the Preferred Reporting Items for Systematic Reviews and Meta-Analyses guidelines (12) (see Supplementary Table 1). Details of the protocol for this analysis were registered (CRD42021283433) on the online database, International Prospective Register of Systematic Reviews. We only included RCTs evaluating MIS approaches for spontaneous ICH to minimize the selection and confounding biases of prospective observational studies. The study groups were defined by randomized assignment to either MIS for ICH evacuation or non-MIS, including medical therapy and conventional craniotomy. We reviewed the methodology of each study to determine which MIS technique was used. If surgery was defined as minimally invasive by the authors, it was included in the MIS group. If an endoscope was used during the evacuation, the technique was categorized as MIS (endoscopic surgery). If a device or catheter was stereotactically placed for infusion of a thrombolytic agent and drainage of the hematoma beyond the time of the operative procedure, the technique was categorized as MIS (stereotactic thrombolysis). The pre-specified primary outcomes were death, defined as a modified Rankin Scale score of 6 or Barthel Index of <30 when the modified Rankin Scale was not available. The analyzed outcome measures were those used in the original studies, with some variability in the scale used to dichotomize the good from the poor outcomes. The modified Rankin Scale and the Barthel Index have high reproducibility and are commonly used to assess neurological outcomes in the ICH.

### Study Selection

PubMed, EMBASE, Web of Science, Ovid, China National Knowledge Infrastructure, and ClinicalTrials.gov were searched for related studies that were either published or established before May 1, 2021. Terms related to “minimally invasive surgery,” “minimally endoscopic surgery,” “minimally stereotactic surgery,” etc., were also searched; no language restriction was applied. The exact search strategy and rationale are shown in Supplementary Material. We obtained additional articles using the reference lists of the articles which were identified in the initial searches. The inclusion criteria were: (1) computed tomography-confirmed diagnosis of spontaneous ICH, and (2) RCTs comparing MIS techniques with other treatment options, including conventional medical treatment and craniotomy. The exclusion criteria were: (1) traumatic brain injury, hemorrhagic tumor, coagulopathy, intracranial aneurysm, cerebral arteriovenous malformation, subdural hemorrhage, epidural hemorrhage, subarachnoid hemorrhage, or pituitary apoplexy; (2) infratentorial ICH, including cerebellar hemorrhage or brain stem hemorrhage; and (3) a total study quality assessment score of < 2. The quality assessment of the study was based on the Cochrane criteria: (1) random sequence generation (yes = 2, unclear = 1, and no = 0); (2) allocation concealment (yes = 2, unclear = 1, and no = 0); (3) blinding of outcome assessment (yes = 2, unclear = 1, and no = 0); and (4) incomplete outcome data reported (yes = 1 and no = 0) (see Supplementary Table 2).

### Study Assessments

Two authors independently identified the articles using the inclusion and exclusion criteria, and the study quality assessment scores were assigned. Any disagreements were resolved by consensus with a third investigator (Dr. Wu).

First, we compared the outcomes between MIS and other treatments. Second, we performed subgroup analyses according to the follow-up duration. We also conducted subgroup analyses focusing on short-term or long-term outcomes, as well as the different interventions.

### Statistical Analysis

The primary outcome of the study was analyzed as categorical variables, with the effectiveness of different treatment methods evaluated and interpreted using a summary odds ratio (OR) and the corresponding 95% confidence interval (CI). Classic χ^2^ test, Q^2^, and I^2^ statistics were used to assess the existence and the magnitude of the between-study heterogeneity. The significance level was set at *p* < 0.05. We used a random-effects model in the analysis. We assumed *a priori* that the meta-analysis could be affected by study-level variability determined by different inclusion criteria across RCTs, which is more appropriately addressed by a random-effects model over a fixed-effects model.

Inverted funnel plots and a regression test were used to assess the potential presence of publication bias. All data analyses were conducted and verified by both R (Version 4.0.0) and RevMan5.4 (Cochrane Information Management System, London, UK).

## Results

### Literature Research

**Figure 1:**
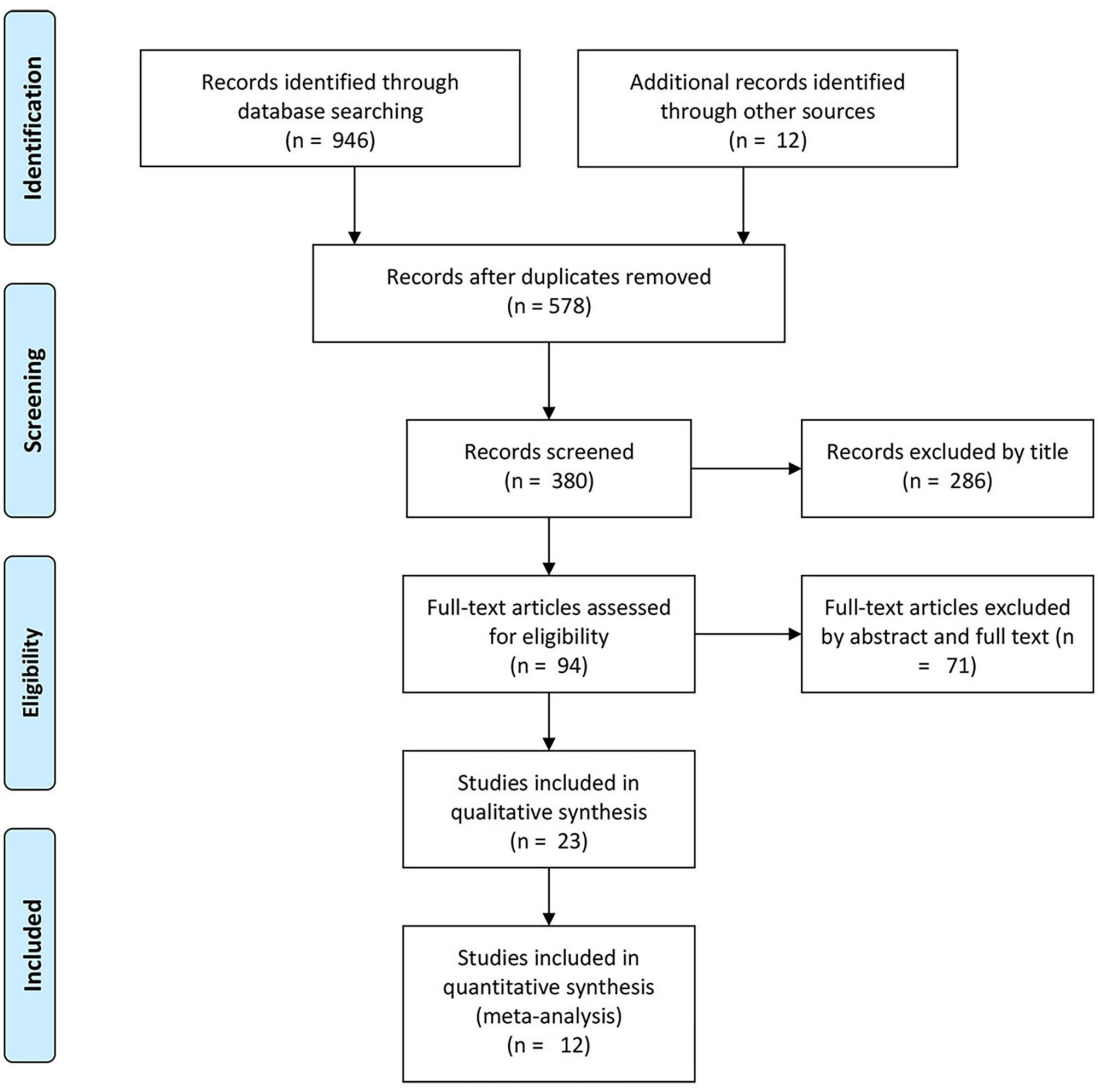
A flow chart of the study collection.

The initial comprehensive literature search identified 946 potentially relevant articles from the databases. A total of 578 studies were excluded as duplicates, leaving a total of 380 studies. Based on the inclusion and exclusion criteria, 286 articles were excluded. Next, we reviewed the full text of the remaining 94 studies, and 71 studies were eliminated after reading the abstracts and full texts. Finally, 11 studies were included in this meta-analysis (see Figure 1 and Table 1).

**Table 1:** Characteristics of the related studies including RCTs and cohorts.

**References**	**Duration**	**Trial type**	**Disease type**	**Treatment methods**				**Hematoma volume (ml)**		**Hematoma volume changes (%)**		**Outcome**		
				**MIS**	**Data**	**Conservative therapy**	**Data**	**MIS**	**Conservative therapy**	**MIS**	**Conservative therapy**	**Outcome**	**MIS numbers**	**Conservative therapy numbers**
Hanley et al. (8)	2006–2013	RCT	ICH	MIS+ rtPA	54	Standard	42	48.2	43.1	–	–	180-day mRS	14	11
Wang et al. (13)	2003–2004	RCT	ICH	MIS	195	Medication	182	33.8	31.3	–	–	3-month BI	19/181	22/165
Auer et al. (14)	1983–1986	RCT	ICH	Endoscopy	50	Medication	50	>50ml: 22; <50ml: 28	>50ml: 24; <50ml: 26	–	–	6-month mRS	42%	70%
Zhou et al. (15)	2005–2008	RCT	ICH	MIS	90	Craniotomy	78	30–100	30–100	–	–	1-year fatality	17	19
Sun et al. (16)	2003–2005	RCT	ICH	Craniopuncture +urokinase	159	Craniotomy	145	52.3	51.7	–	–	90-day BI	29	26
Kim and Kim (17)	2001–2009	RCT	ICH	Stereotactic	204	Craniotomy	183	24	21	–	–	6-month mortality	11	7
Hattori et al. (18)	1998–2000	RCT	ICH	Stereotactic	121	Conservative	121	–	–	–	–	Mortality	11.8%	23.5%
Zuccarello et al. (19)	1994–1996	RCT	ICH	Stereotactic	4	Medication	11	35	30	44%	0	3-month BI	0	3
Vespa et al. (20)	2009–2012	RCT	ICH	Endoscopy	14	Medical	39	38	40	25	3	30-day mortality	2	9
Vespa et al. (20)	2009–2012	RCT	ICH	Surgery	13	Medical	26	38	40	25	3	1-year mortality	15%	40%
Teernstra et al. (21)	1996–1999	RCT	ICH	Surgery	36	Non-surgery	34	66	52	17.9	7	180-day mRS	20	20
Yang et al. (22)	2012–2014	RCT	ICH	MIS	78	Craniotomy	78	–	–	45	75	12^th^ week BI	3	19
Feng et al. (23)	2006–2013	RCT	ICH	Keyhole	93	Craniotomy	91	–	–	–	–	6-month ADL	6	8
Sun et al. (16)	2015–2016	Cohort	ICH	Keyhole	46	Craniotomy	43	–	–	95%	82%	6-month mortality	4.3%	4.7%

*RCT, randomized controlled trial; ICH, intracerebral hemorrhage; MIS, minimally invasive surgery; mRS, modified Rankin scale; BI, Barthel index; rtPA, recombinant tissue plasminogen activator; ADL, activity of daily living*.

### Comparisons Between the MIS and Control Groups for Mortality and Rebleeding Morbidity

**Figure 2:**
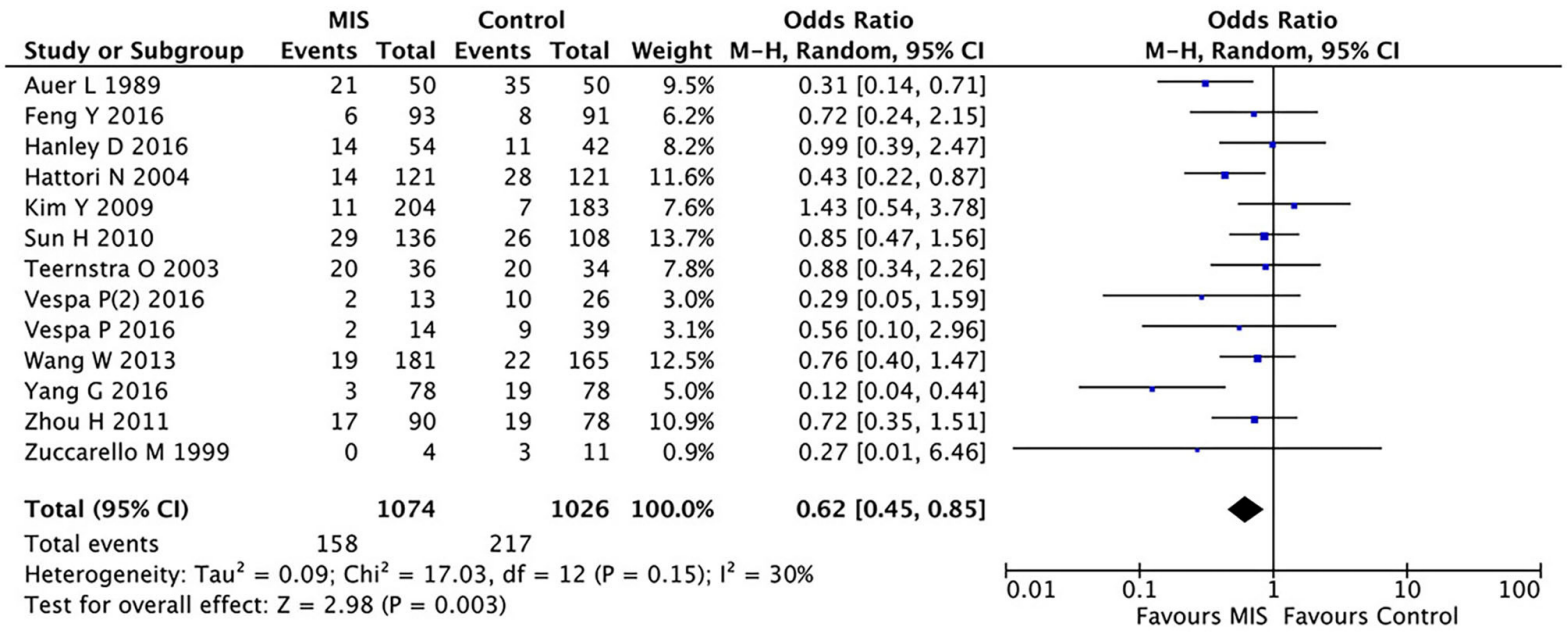
A forest plot of the minimally invasive surgery and control groups for overall mortality.

In 12 RCTs (8, 13–23) involving 2,100 patients, the MIS group had decreased the short-term or long-term mortality (OR = 0.62, 95% CI 0.45–0.85; I^2^ = 30%, *P* = 0.15) compared to the control group (i.e., medication or open surgery). This suggests that MIS can effectively reduce the postoperative death rate (see Figure 2). For rebleeding after the treatment, we included five RCTs (Sun H, Teernstra O, Wang W, Hanley D, and Vespa P) into the analysis and found no significant differences between the MIS and the control groups (OR = 1.86, 95% CI 0.47–7.36; I^2^ = 78%, *P* = 0.001, see Supplementary Figure 5). We then removed the data from Vespa, which probably caused heterogeneity from the original analysis, performed the statistical analysis again (not shown), and found that the heterogeneity of the data did not decrease significantly (I^2^ = 83%, *P* = 0.004; OR = 1.93, 95% CI 0.42–8.91), which suggests that the determining factor leading to heterogeneity is not the presence or the absence of the study by Vespa but, probably, the different internal designs of each study.

### Subgroup Analysis Between the MIS and the Control Groups for Long-Term Mortality

**Figure 3:**
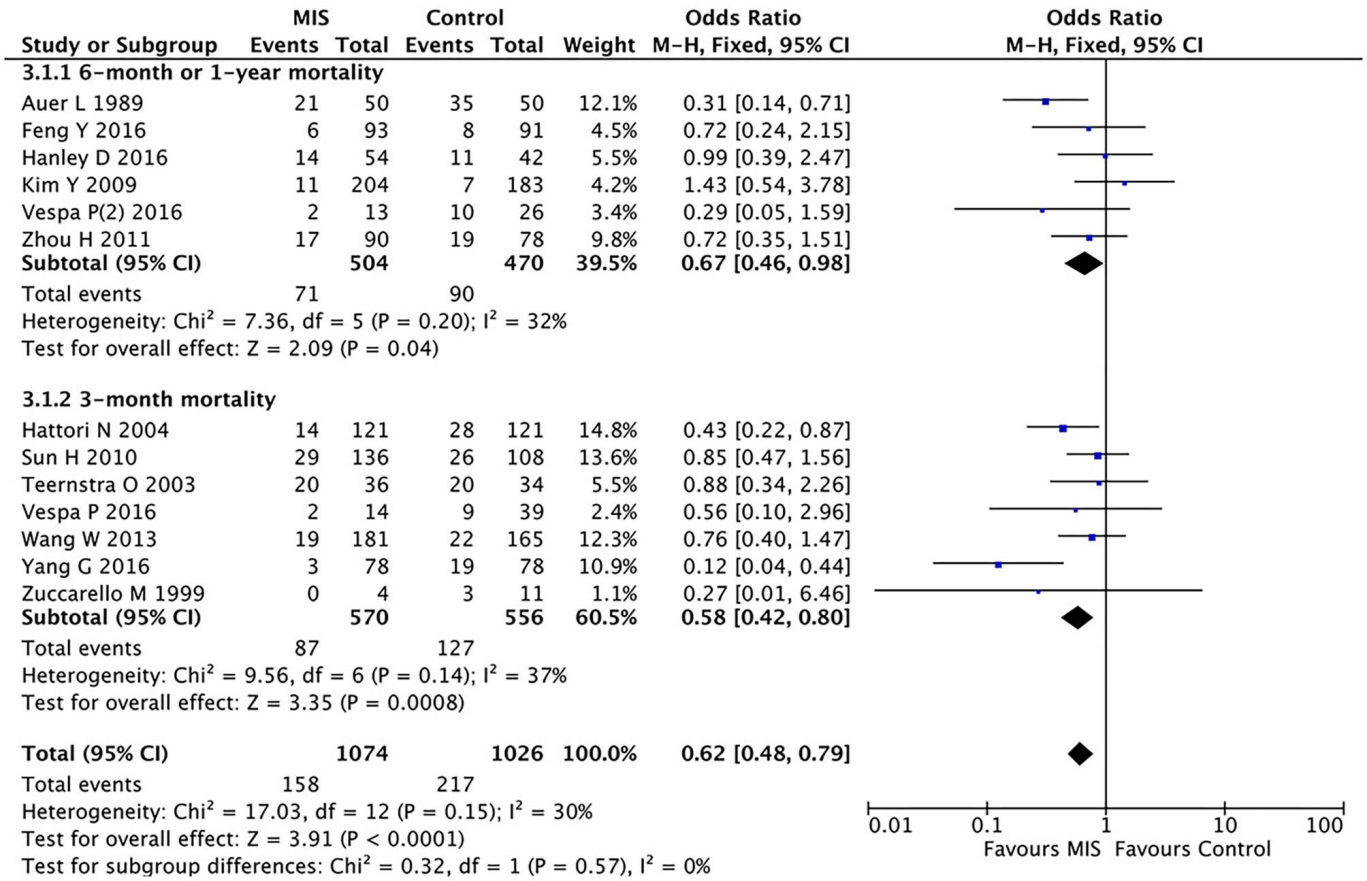
A forest plot of the subgroup analysis of the minimally invasive surgery and control groups for long-term (6-month or 1-year) and short-term (3-month) mortality.

In five RCTs involving 974 patients, the MIS group had decreased the long-term (6-month or 1-year) mortality (OR = 0.67, 95% CI 0.46–0.98; I^2^ = 32%, *P* = 0.20) compared to the control group (i.e., medication or open surgery). This suggests that MIS can effectively reduce the long-term postoperative death rate (see Figure 3).

### Subgroup Analysis Between the MIS and Control Groups for Short-Term Mortality

In eight RCTs involving 1,126 patients (Figure 3), the MIS group had decreased the short-term (3-month) mortality (OR = 0.58, 95% CI 0.42–0.80; I^2^ = 37%, *P* = 0.14) compared to the control group (i.e., medication or open surgery). This suggests that MIS can effectively reduce the short-term postoperative death rate. Thus, MIS is beneficial for both long-term and short-term prognoses.

### Comparisons Between the MIS and Control Groups for Hematoma Evacuation

**Figure 4:**
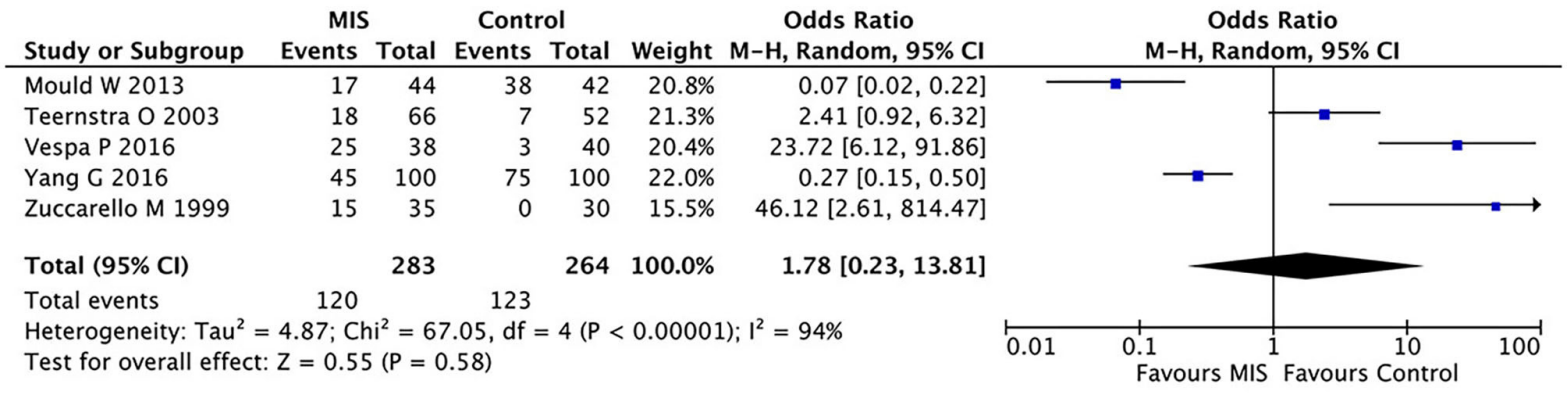
A forest plot of the hematoma evacuation rate.

As most studies did not report hematoma clearance rates, this comparison is limited to the data reported in five studies. In these five RCTs involving 404 patients, the MIS group had no significant effect on mortality compared to the control group (OR = 0.36, 95% CI 0.06–2.13; I^2^ = 91%, *p* < 0.00001) as shown in Figure 4.

### Subgroup Analysis Between the MIS and Craniotomy Groups for Mortality

**Figure 5:**
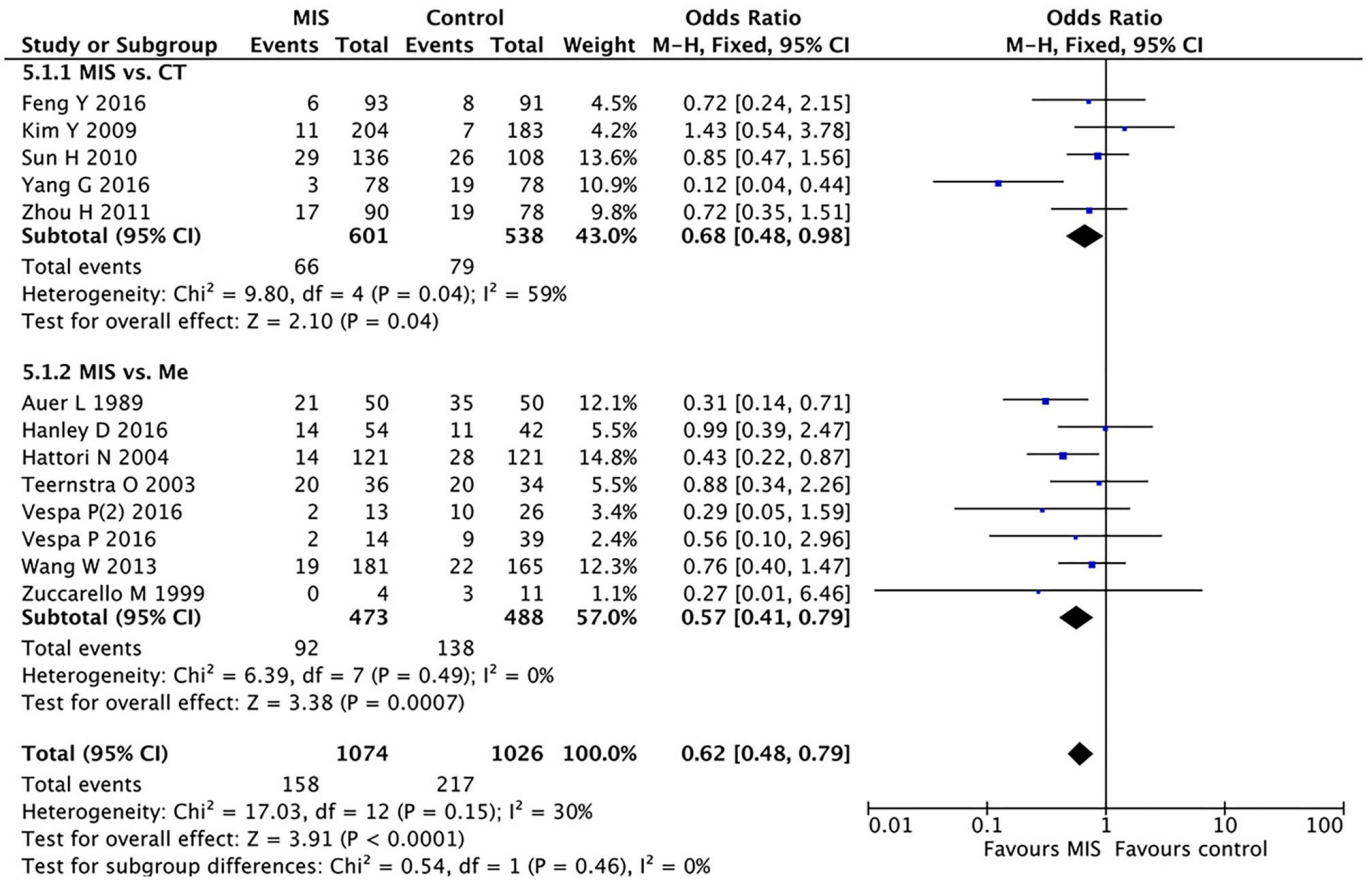
A forest plot of the subgroup analysis of the minimally invasive surgery (MIS) and craniotomy and MIS and medication groups for overall mortality.

We divided the control group into two subgroups: one for patients undergoing craniotomy and one for those treated with conventional drugs. We analyzed the two subgroups separately. In five RCTs involving 1,139 patients, the MIS group had insignificantly decreased the short-term or long-term mortality compared to open craniotomy as shown in Figure 5. The OR was 0.68 (95% CI 0.48–0.98) with large heterogeneity (I^2^ = 59%, *P* = 0.04).

### Subgroup Analysis Between the MIS and Medication Groups for Mortality

In eight RCTs involving 961 patients, the MIS group had decreased the short-term or long-term mortality compared to conservative medication (OR = 0.57, 95% CI 0.41–0.79; I^2^ = 0%, *P* = 0.49), as shown in Figure 5.

### Publication Bias and Egger's Test

Egger's test results for mortality between the MIS and the control groups (z = −0.4950, *P* = 0.6206) in the general population suggested that the publication bias across the included studies was unlikely.

## Discussion

In this systematic review and meta-analysis, we have incorporated recent data to reevaluate the total effect of MIS in treating ICH, as well as the effect on mortality by using the MIS techniques, including endoscopic surgery and stereotactic thrombolysis. We found that MIS techniques have independently decreased the rate of death at the short-term follow-up. In addition, we performed subgroup analyses focusing on different control groups, demonstrating that MIS techniques is potentially beneficial compared with conservative medication and open craniotomy. However, another subgroup analysis focusing on different follow-up durations demonstrated that MIS techniques seem beneficial at the 3-month follow-up and at the 6-month or 1-year follow-up.

Study design, including the primary and secondary outcome measures and the subgroup analyses, were modeled after previous meta-analyses on this topic, as performed by Prasad, Zhou et al. (11), and Scaggiante et al. (10) to permit several comparisons, including the comparison of MIS and conventional surgical efficacy, comparisons between different populations, Glasgow coma scale (GCS) scores, and different hematoma volumes. Previous studies have demonstrated that “patients with supratentorial ICH may benefit more from MIS than other treatment options. The most likely candidates to benefit from MIS are both sexes, those aged between 30 and 80 years with superficial hematoma, a GCS score of ≥ 9, hematoma volume between 25 and 40 ml, and MIS performed within 72 h after the onset of symptoms” (11), and “endoscopic surgery and stereotactic thrombolysis seemed to independently decrease the rate of moderate to severe functional impairment and death at the long-term follow-up” (10). The main aim of this study was to investigate the relationship between MIS techniques on the short-term, long-term, and overall prognosis of patients with ICH, as well as to observe the effect of MIS vs. massively invasive surgery, and MIS vs. conventional drug treatment on the overall prognosis.

The effect of MIS and craniotomy on overall prognosis (mortality) was not statistically significant, meaning that the size of the surgical trauma did not determine the mortality of the patient, a point that has rarely been studied. However, MIS reduces overall mortality in patients with ICH compared with massively invasive surgery, suggesting that MIS has advantages. Hanley et al. (8) studied the postoperative complications in patients with ICH and found that the combination of the MIS and a thrombolytic drug therapy was not effective in reducing the risk of postoperative infection and rebleeding when compared with standard drug therapy. In addition, Mendelow et al. (6) studied the age, GCS score, size, and location of the hematoma before surgery and found no advantage of an early massively invasive surgery over conventional drug therapy in these areas. Zhou et al. found the superiority of MIS techniques in the abovementioned areas to improve the prognosis of the patient (11). Advanced age (>80 years), low GCS score, and hematoma volume > 30 ml are all key factors that exacerbate the prognosis of patients with ICH (1).

Our results also suggest that MIS effectively reduces mortality at 6-month or 1-year follow-up when compared with non-MIS treatment, and it was also effective in reducing the mortality at a 3-month follow-up. This suggests that MIS mainly affects the short-term and long-term prognosis of patients. Owing to the lack of sufficient data, no statistical significance was found when comparing the MIS technique with debridement surgery in terms of hematoma clearance rates. Our study illustrates, to some extent, the impact of MIS on the prognosis of patients with ICH, mainly affecting the short-term prognosis, and that this impact of MIS on prognosis may be accomplished by selecting the appropriate population for treatment.

Meta-analyses are classically limited by the heterogeneity of the inclusion and exclusion criteria of the examined studies, as well as the variability of the outcome measures. In these studies, the evaluations of death varied, such as in Zhang et al. in which death was defined as a modified Rankin Scale score of 6, and in Yang and Shao (22) in which functional outcomes were defined using the Barthel Index. In certain cases, the outcomes for both primary and secondary outcomes were not available. Therefore, the conclusions of this meta-analysis are limited by the quality and the heterogeneity of the data. Although not perfectly homogenous, many studies permit quantitative analyses that may inform future trial design and clinical practice.

## Conclusions

We found that MIS techniques independently decreased the death rate at short-term and long-term follow-ups. In addition, we performed subgroup analyses, demonstrating that MIS techniques seem beneficial compared with conservative medication.

## Data Availability Statement

The original contributions presented in the study are included in the article/Supplementary Material, further inquiries can be directed to the corresponding author/s.

## Author Contributions

QD, DW, and YT designed this project and proofread and reviewed the manuscript. DH and YL reviewed the articles and collected data. DH, DW, and YT analyzed data. DH drafted the manuscript and polished the final manuscript. All authors contributed to the article and approved the submitted version.

## Funding

The present study was supported by grants from the Minhang District Health Bureau of Shanghai (Grant No. 2020MWDXK01), and Shanghai Fifth People's Hospital (Grant No. 2020WYZDZK04).

## Conflict of Interest

The authors declare that the research was conducted in the absence of any commercial or financial relationships that could be construed as a potential conflict of interest.

## Publisher's Note

All claims expressed in this article are solely those of the authors and do not necessarily represent those of their affiliated organizations, or those of the publisher, the editors and the reviewers. Any product that may be evaluated in this article, or claim that may be made by its manufacturer, is not guaranteed or endorsed by the publisher.
