# Supplementary material for: Minimally Invasive Surgery in Patients With Intracerebral Hemorrhage: A Meta-Analysis of Randomized Controlled Trials
Source: Front Neurol. 2022 Jan 13;12:789757. doi: 10.3389/fneur.2021.789757 (PMC8793625; doi:10.3389/fneur.2021.789757)
Supplement: Supplementary file 1 [file Data_Sheet_1.DOCX]

**SUPPLEMENTARY MATERIALS**

**Minimally invasive surgery in patients with intracerebral hemorrhage: a meta-analysis of randomized controlled trials**

Duanlu Hou^1^, Ying Lu^2^, Danhong Wu^1^, Yuping Tang^1,2^, Qiang Dong^2^

1. Department of Neurology, Shanghai Fifth People’s Hospital, Fudan University, Shanghai, China

2. Department of Neurology, Huashan Hospital, Fudan University, Shanghai, China

**Searching strategy**

Pubmed

# 1 Search: ((minimally stereotactic surgery) AND (intracerebral hemorrhage)) AND (randomized controlled trial)

"minimally"[All Fields] AND ("stereotactic"[All Fields] OR "stereotactical"[All Fields] OR "stereotactically"[All Fields] OR "stereotactics"[All Fields]) AND ("surgery"[MeSH Subheading] OR "surgery"[All Fields] OR "surgical procedures, operative"[MeSH Terms] OR ("surgical"[All Fields] AND "procedures"[All Fields] AND "operative"[All Fields]) OR "operative surgical procedures"[All Fields] OR "general surgery"[MeSH Terms] OR ("general"[All Fields] AND "surgery"[All Fields]) OR "general surgery"[All Fields] OR "surgery s"[All Fields] OR "surgerys"[All Fields] OR "surgeries"[All Fields]) AND ("intracerebral haemorrhage"[All Fields] OR "cerebral hemorrhage"[MeSH Terms] OR ("cerebral"[All Fields] AND "hemorrhage"[All Fields]) OR "cerebral hemorrhage"[All Fields] OR ("intracerebral"[All Fields] AND "hemorrhage"[All Fields]) OR "intracerebral hemorrhage"[All Fields]) AND ("randomized controlled trial"[Publication Type] OR "randomized controlled trials as topic"[MeSH Terms] OR "randomized controlled trial"[All Fields] OR "randomised controlled trial"[All Fields])

# 2 Search: ((minimally invasive surgery) AND (intracerebral hemorrhage)) AND (randomized controlled trial)

("minimally invasive surgical procedures"[MeSH Terms] OR ("minimally"[All Fields] AND "invasive"[All Fields] AND "surgical"[All Fields] AND "procedures"[All Fields]) OR "minimally invasive surgical procedures"[All Fields] OR ("minimally"[All Fields] AND "invasive"[All Fields] AND "surgery"[All Fields]) OR "minimally invasive surgery"[All Fields]) AND ("intracerebral haemorrhage"[All Fields] OR "cerebral hemorrhage"[MeSH Terms] OR ("cerebral"[All Fields] AND "hemorrhage"[All Fields]) OR "cerebral hemorrhage"[All Fields] OR ("intracerebral"[All Fields] AND "hemorrhage"[All Fields]) OR "intracerebral hemorrhage"[All Fields]) AND ("randomized controlled trial"[Publication Type] OR "randomized controlled trials as topic"[MeSH Terms] OR "randomized controlled trial"[All Fields] OR "randomised controlled trial"[All Fields])

# 3 Search: ((minimally endoscopic surgery) AND (intracerebral hemorrhage)) AND (randomized controlled trial)

"minimally"[All Fields] AND ("endoscopy"[MeSH Terms] OR "endoscopy"[All Fields] OR ("endoscopic"[All Fields] AND "surgery"[All Fields]) OR "endoscopic surgery"[All Fields]) AND ("intracerebral haemorrhage"[All Fields] OR "cerebral hemorrhage"[MeSH Terms] OR ("cerebral"[All Fields] AND "hemorrhage"[All Fields]) OR "cerebral hemorrhage"[All Fields] OR ("intracerebral"[All Fields] AND "hemorrhage"[All Fields]) OR "intracerebral hemorrhage"[All Fields]) AND ("randomized controlled trial"[Publication Type] OR "randomized controlled trials as topic"[MeSH Terms] OR "randomized controlled trial"[All Fields] OR "randomised controlled trial"[All Fields])

# 4 Search: ((((minimally endoscopic surgery) AND (intracerebral hemorrhage)) AND (randomized controlled trial)) AND (((minimally stereotactic surgery) AND (intracerebral hemorrhage)) AND (randomized controlled trial))) AND (((minimally invasive surgery) AND (intracerebral hemorrhage)) AND (randomized controlled trial))

"minimally"[All Fields] AND ("endoscopy"[MeSH Terms] OR "endoscopy"[All Fields] OR ("endoscopic"[All Fields] AND "surgery"[All Fields]) OR "endoscopic surgery"[All Fields]) AND ("intracerebral haemorrhage"[All Fields] OR "cerebral hemorrhage"[MeSH Terms] OR ("cerebral"[All Fields] AND "hemorrhage"[All Fields]) OR "cerebral hemorrhage"[All Fields] OR ("intracerebral"[All Fields] AND "hemorrhage"[All Fields]) OR "intracerebral hemorrhage"[All Fields]) AND ("randomized controlled trial"[Publication Type] OR "randomized controlled trials as topic"[MeSH Terms] OR "randomized controlled trial"[All Fields] OR "randomised controlled trial"[All Fields]) AND ("minimally"[All Fields] AND ("stereotactic"[All Fields] OR "stereotactical"[All Fields] OR "stereotactically"[All Fields] OR "stereotactics"[All Fields]) AND ("surgery"[MeSH Subheading] OR "surgery"[All Fields] OR "surgical procedures, operative"[MeSH Terms] OR ("surgical"[All Fields] AND "procedures"[All Fields] AND "operative"[All Fields]) OR "operative surgical procedures"[All Fields] OR "general surgery"[MeSH Terms] OR ("general"[All Fields] AND "surgery"[All Fields]) OR "general surgery"[All Fields] OR "surgery s"[All Fields] OR "surgerys"[All Fields] OR "surgeries"[All Fields]) AND ("intracerebral haemorrhage"[All Fields] OR "cerebral hemorrhage"[MeSH Terms] OR ("cerebral"[All Fields] AND "hemorrhage"[All Fields]) OR "cerebral hemorrhage"[All Fields] OR ("intracerebral"[All Fields] AND "hemorrhage"[All Fields]) OR "intracerebral hemorrhage"[All Fields]) AND ("randomized controlled trial"[Publication Type] OR "randomized controlled trials as topic"[MeSH Terms] OR "randomized controlled trial"[All Fields] OR "randomised controlled trial"[All Fields])) AND (("minimally invasive surgical procedures"[MeSH Terms] OR ("minimally"[All Fields] AND "invasive"[All Fields] AND "surgical"[All Fields] AND "procedures"[All Fields]) OR "minimally invasive surgical procedures"[All Fields] OR ("minimally"[All Fields] AND "invasive"[All Fields] AND "surgery"[All Fields]) OR "minimally invasive surgery"[All Fields]) AND ("intracerebral haemorrhage"[All Fields] OR "cerebral hemorrhage"[MeSH Terms] OR ("cerebral"[All Fields] AND "hemorrhage"[All Fields]) OR "cerebral hemorrhage"[All Fields] OR ("intracerebral"[All Fields] AND "hemorrhage"[All Fields]) OR "intracerebral hemorrhage"[All Fields]) AND ("randomized controlled trial"[Publication Type] OR "randomized controlled trials as topic"[MeSH Terms] OR "randomized controlled trial"[All Fields] OR "randomised controlled trial"[All Fields]))

SF1: Funnel plot of the minimally invasive surgery and control groups for overall mortality

SF2: Funnel plot of the subgroup analysis of the minimally invasive surgery and control groups for long-term (6-month or 1-year) and short-term (3-month) mortality

SF3: Funnel plot of the hematoma evacuation rate

SF4: Funnel plot of the subgroup analysis of the minimally invasive surgery (MIS) and craniotomy and MIS and medication groups for overall mortality

SF5: Forest plot of the minimally invasive surgery and control groups for re-bleeding morbidity
