# Supplementary material for: Minimally Invasive Surgery in Patients With Intracerebral Hemorrhage: A Meta-Analysis of Randomized Controlled Trials
Source: Front Neurol. 2022 Jan 13;12:789757. doi: 10.3389/fneur.2021.789757 (PMC8793625; doi:10.3389/fneur.2021.789757)
Supplement: Supplementary file 3 [file Table_2.DOCX]

| Jadad scale | | | | | |
| --- | --- | --- | --- | --- | --- |
|  | Randomized generation | Outcome blinding | Incomplete data | Allocation concealment | Total score |
| Hanley | 2 | 2 | 1 | 2 | 7 |
| Vespa | 1 | 2 | 1 | 1 | 5 |
| Auer | 1 | 0 | 1 | 0 | 2 |
| Zuccarello | 2 | 0 | 1 | 2 | 5 |
| Teernstra | 2 | 0 | 1 | 2 | 5 |
| Hattori | 2 | 2 | 1 | 2 | 7 |
| Kim | 1 | 0 | 1 | 2 | 4 |
| Wang | 2 | 2 | 1 | 2 | 7 |
| Sun | 1 | 2 | 1 | 2 | 6 |
| Zhou | 2 | 0 | 1 | 0 | 3 |
| Zhang | 1 | 1 | 1 | 2 | 5 |
| Feng | 1 | 1 | 1 | 1 | 4 |
